# X-ray Microtomography to Assess Determinants of In Vivo N-Butyl Cyanoacrylate Glubran^®^2 Polymerization: A Rabbit-Model Study

**DOI:** 10.3390/biomedicines10102625

**Published:** 2022-10-19

**Authors:** Kévin Guillen, Pierre-Olivier Comby, Anne-Virginie Salsac, Nicolas Falvo, Marc Lenfant, Alexandra Oudot, Hugo Sikner, Anne Dencausse, Emilie Laveissiere, Serge Ludwig Aho-Glele, Romaric Loffroy

**Affiliations:** 1 Department of Vascular and Interventional Radiology, Image-Guided Therapy Center, François-Mitterrand University Hospital, 14 Rue Paul Gaffarel, BP 77908, 21079 Dijon, France; 2 Imaging and Artificial Vision (ImViA) Laboratory-EA 7535, Bourgogne/Franche-Comté University, 9 Avenue Alain Savary, BP 47870, 21078 Dijon, France; 3 Department of Neuroradiology and Emergency Radiology, François-Mitterrand University Hospital, 14 Rue Paul Gaffarel, BP 77908, 21079 Dijon, France; 4 Biomechanics and Bioengineering Laboratory, UMR CNRS 7338, Université de Technologie de Compiègne, 60203 Compiègne, France; 5 Department of Nuclear Medicine, Plateforme d’Imagerie et de Radiothérapie Préclinique, Georges-François Leclerc Center, 1 Rue Professeur Marion, BP 77980, CEDEX, 21079 Dijon, France; 6 R&D, Guerbet Research, CEDEX, 95943 Roissy Charles-de-Gaulle, France; 7 Department of Epidemiology, Statistics and Clinical Research, François-Mitterrand University Hospital, 14 Rue Paul Gaffarel, BP 77908, 21079 Dijon, France

**Keywords:** 3D imaging, X-ray microtomography, cyanoacrylate, in vivo, lipiodol

## Abstract

Although introduced decades ago, few cyanoacrylate glues have been approved for endovascular use, despite evidence of their usefulness, notably for complex procedures suchas hemostatic embolization. Indications include massive bleeding requiring emergent hemostasis and prevention of severe bleeding during scheduled surgery to remove a hypervascular tumor. Adding radiopaque Lipiodol Ultra Fluid^®^ (LUF) modulates glue polymerization and allows fluoroscopic guidance, but few comparative in vivo studies have assessed the impact of the resulting change in glue concentration or of other factors such as target-vessel blood flow. In a rabbit model, we used ex vivo X-ray microtomography to assess the results of in vivo renal-artery embolization by various mixtures of N-butyl cyanoacrylate (NBCA), metacryloxysulfolane, and LUF. Overall, penetration to the superficial interlobular arteries was achieved in about two-thirds of cases and into the capillaries in nearly half the cases, while cast fragmentation was seen in slightly more than half the cases. Greater NBCA dilution and the blocked-blood-flow technique were independently associated with greater distality of penetration. Blocked-blood-flow injection was independently associated with absence of fragmentation, capillary penetration, a shorter cast-to-capsule distance, and higher cast attenuation. A larger mixture volume was independently associated with higher indexed cast ratio and deeper penetration. Finally, microtomography is an adapted tool to assess ex vivo distribution of glue cast.

## 1. Introduction

Embolization is among the minimally invasive endovascular procedures commonly performed by interventional radiologists. Compared to surgery, endovascular embolization induces fewer complications, does not require general anesthesia, and can often be done on a day-care basis, which decreases costs [1]. Transcatheter embolization is used to treat vessel lesions due to disease or injury, notably when bleeding occurs. A wide variety of embolic agents including metallic coils, calibrated microspheres, and liquids are available in clinical practice. The spectrum of highly prevalent health conditions for which liquid embolic agents including N-butyl cyanoacrylate (NBCA) have been proven useful is expanding steadily and now includes lower urinary-tract symptoms due to prostate enlargement, aneurysms and pseudoaneurysms, arteriovenous malformations, other vascular lesions responsible for bleeding, and solid tumors [2,3,4,5,6,7,8]. NBCA glues have been extensively studied since the late 1980s. Nonetheless, the factors that affect NBCA polymerization into a solid cast after intravascular injection remain incompletely elucidated [9]. For distal vascular lesions, mechanical devices are not appropriate. However, although liquid agents penetrate distally, their premature or excessively proximal polymerization may be followed by recanalization via the development of collaterals [10]. Moreover, the smaller the embolized vessel, the greater the likelihood of organ ischemia and of mixture reflux with non-targetted embolization, which is among the main risk factors for potentially fatal complications such as healthy-tissue ischemia [11]. Additionally, NBCA polymerization can cause catheter-lumen occlusion or catheter adhesion to the vascular intima [12]. Thus, operators need detailed information allowing them to control the polymerization of each NBCA product by adjusting factors such as glue dilution and injection volume, notably when treating complex abnormalities such as arteriovenous malformations. To date, in clinical practice, the glue concentration in the embolizing mixture and the injection rate are adjusted empirically to ensure that polymerization occurs at the target site. This adjustment requires extensive operator training and navigation of a potentially long and tricky learning curve [4,13,14,15,16]. Existing methods used to control NBCA polymerization include the pressure-cooker or blocked-blood-flow (BBF) technique, in which a second microcatheter occludes the vessel proximally to prevent the reflux and fragmentation of the glue injected distally through the first microcatheter [17]. Moreover, the polymerization rate can be slowed by decreasing the NBCA concentration in the embolic mixture via the addition of lipiodol (Lipiodol Ultra Fluid^®^ [LUF], Guerbet, Villepinte, France) [15,18,19]. The slower rate gives the operator more leeway, and the radiopacity of lipiodol enables fluoroscopic monitoring [19]. Anhydrous ethanol (AE) also delays endothelial adhesion by increasing interface polymerization and cohesive properties of NBCA [20,21]. Glubran^®^2 (GEM, Viareggio, Italy) is NBCA combined with the monomer metacryloxysulfolane (MS) to improve stability and decrease the in vivo exothermic reaction. It was the first cyanoacrylate glue to receive the European Union certification mark for endovascular use. Nonetheless, few studies have focused on the specific polymerization characteristics of Glubran^®^2 to better anticipate its in vivo behavior [15,22]. We therefore chose to use Glubran^®^2 for an in vivo animal study with the objective of assessing glue distribution in the downstream vascular network and parenchymal penetration using ex vivo X-ray microtomography (micro-CT).

## 2. Materials and Methods

### 2.1. Experimental Model and Procedure

**Figure 1:**
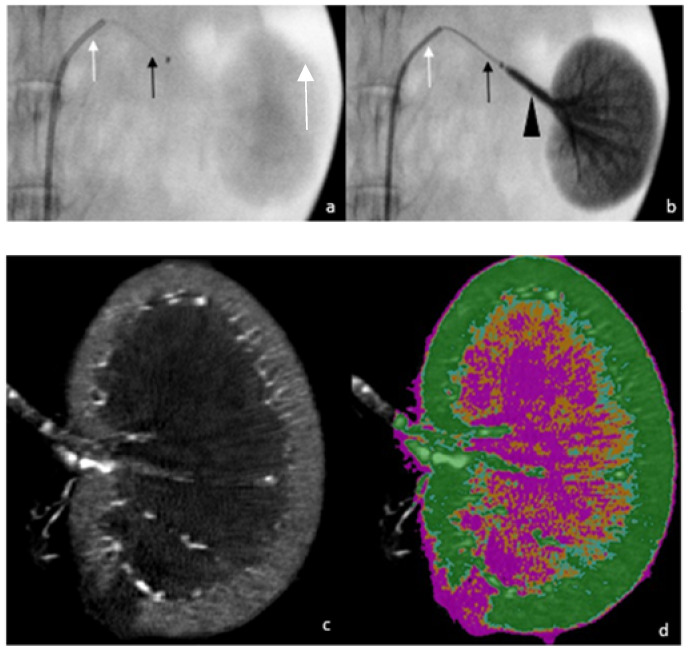

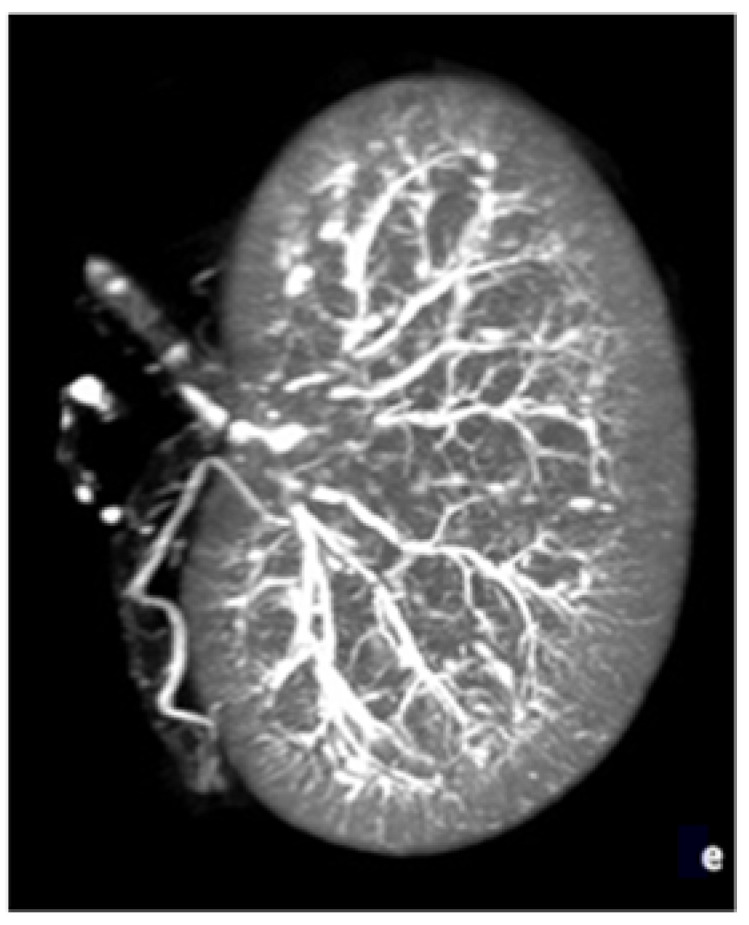
Example of a procedure, from embolization under fluoroscopic guidance to micro-CT imaging. Fluoroscopic view before (**a**) and after (**b**) embolization, with the white arrow showing the 4-Fr vertebral catheter in the left renal artery, the black arrow showing the 2.3-Fr microcatheter, and the black arrowhead showing cast formation during the injection. Coronal ex vivo micro-CT images before (**c**) and after (**d**) post-processing segmentation, with colors on the d view as follows: pink, renal parenchyma segmentation; orange, cast segmentation from 1000 to 1300 HU; cyan blue, cast segmentation from 1300 to 1600 HU; and green, cast segmentation above 1600 HU. (**e**) Maximum intensity projection reconstruction, used for a more detailed assessment of fragmentation.

We performed the experiments on the renal arteries of female rabbits. These vessels are suitable to our objective of assessing downstream distribution and parenchymal penetration because it is a terminal vascular network with no extra renal anastomosis. The animal experiments complied with ARRIVE 2.0 guidelines on the protection of animals used for scientific purposes, and the study protocol was approved by the local animal-experiment ethics committees (APAFIS number: #25592 committee 106). We used 26 healthy female rabbits aged about 3 weeks and weighing 2.5 to 4.4 kg. The animals were sedated then anesthetized using 2% isoflurane (Zoetis) under spontaneous respiration. Anesthesia depth and physiological parameters were monitored continuously during the procedure. All experiments were carried out by two radiologists (P.O.C. and K.G.) under fluoroscopic guidance (Veradius, Philips, Amsterdam, The Netherlands). A 4-French (Fr) introducer was inserted through a surgical right-femoral approach. A baseline abdominal angiogram was obtained by injecting a water-soluble contrast agent (Xenetix, Guerbet) into a vertebral 4-Fr diagnostic catheter. The two renal arteries were then catheterized successively using a 2.3-Fr microcatheter (Phenom 21, Medtronic, Tolochenaz, Switzerland) inserted coaxially through the 4-Fr catheter and positioned 1 cm proximal to the first bifurcation of renal artery. The embolization technique was performed as follows: at room temperature, Glubran^®^2 was mixed manually with LUF in a 1:1, 1:3, or 1:7 ratio, with or without AE, using a LUF-resistant three-way stopcock (Vectorio, Guerbet). Before the embolic-mixture injection, the microcatheter was flushed with 5% dextrose to remove any anions potentially responsible for premature glue polymerization. For BBF embolization, after positioning of the injection microcatheter, the vertebral catheter was pushed into the trunk of the target artery to stop blood flow (Figure 1a,b). For embolization with preserved blood flow, the vertebral catheter was left in the aorta. Table 1 shows the numbers of repetitions for each of these two techniques. In total, 42 embolization procedures were performed with Glubran^®^2 (i.e., with NBCA-MS). A standardized injection was performed using an automatic power injector (Harvard PHD 2000 Advance Syringe Pump, Holliston, MA, USA) at a rate of 0.03 mL/s with a 5-mL syringe (Plastipak, Becton Dickinson Plastic, Franklin Lakes, NJ, USA). The flow rate was determined by adapting data from our clinical experience with NBCA embolization to the physical characteristics of the rabbit model, notably regarding vessel diameter. The injection was stopped when glue backflow on the microcatheter tip was detected fluoroscopically, and the microcatheter was immediately retracted to prevent tip adhesion to the vessel wall. The contralateral embolization procedure was performed using a new microcatheter. Some rabbits underwent embolization of the second renal artery with another component. These were excluded from this analysis. The animals were euthanized 30 min after the last embolization, using an intravenous bolus of sodium pentobarbital (Dolethal^®^, Vetoquinol, Lure, France) while still under general anesthesia. The kidneys were harvested and fixed in 4% formaldehyde. The intravascular distribution of the embolic mixture in the kidneys was assessed 2–4 weeks later, using micro-CT. 

### 2.2. Acquisition and Reconstruction Parameters for Micro-CT

We used the micro-CT component of the NanoSPECT/CT Plus small-animal camera (Bioscan, Bioscan Inc., Washington, DC, USA). A scout view was firstly acquired to determine the required axial scanning range. The acquisition voltage was 55 kV, exposure time was 1000 ms for each of the 240 projections, axial range was 40–50 mm, and pitch was 1. Each acquisition lasted 6–9 min. The micro-CT reconstructions were obtained using Bioscan image-processing software. An exact cone-beam filtered back-projection algorithm produced reconstructed image slices with a voxel size of 36 × 36 × 73 µm^3^. A Ram-Lak filter was applied with the cutoff frequency set at the Nyquist frequency. See Figure 1c–e.

### 2.3. Data Analyses and Outcomes

Each reconstructed image was opened using VivoQuant™ software (Invicro, Boston, MA, USA). Each specimen was carefully reoriented before measurement. All measurements were made by the same investigator.

#### 2.3.1. Objective Outcomes

The cast-to-capsule distance (mm) was measured at the upper pole, in the middle, and at the lower pole of the kidney, and the depth of penetration of the mixture was assessed. The embolized kidney areas were segmented, starting at the first renal-artery bifurcation to avoid cast-volume variability due to differences in available artery trunk length after harvesting. In the first segment, the cast was segmented based on whether attenuation was 1000 to 1300 HU, >1300 to 1600 HU, or >1600 HU, where HU is Hounsfield unit. These cutoffs were chosen empirically, considering LUF hyperdense and avoiding constructed hyperdensity with steak artefact. We hypothesized that the higher the density in each volume, the higher the amount of glue. Each segmented cast was normalized on renal-parenchyma segmentation to limit variability due to individual characteristics of each animal (e.g., weight and age). The resulting variable is named indexed cast ratio.

#### 2.3.2. Subjective Outcomes

Semi-quantitative scoring was performed to evaluate the distality of glue distribution, 1 indicating presence in the main renal artery and first branches, 2 in the interlobar artery, 3 at the corticomedullary junction, 4 in the deep cortical interlobular arteries, and 5 in the superficial cortical interlobular arteries. Cast fragmentation was scored 0 if absent and 1 if present: this score represents the ability to control the cast constitution. Fragmentation of the cast is more likely to result in non-target embolization. Cast heterogeneity was scored in the first three distribution areas (1 to 3 above), from 0 to 4 (0%, present in ≤25%, >25% and ≤50%, >50% and ≤75%, and 75% to 100%). We hypothesized that high heterogeneity leads to delayed distal embolization. Uncertainties occurred regarding the assessment of distality and, consequently, only heterogeneity scores in the two most proximal areas were included in the statistical analysis. Finally, increased cortical blurring indicating a capillary penetration was scored 0 if absent and 1 if present to assess the ischemic effect of embolization. 

#### 2.3.3. Image-Quality Criteria

Objective image quality was assessed by drawing two spherical regions of interest (ROIs) in the background of the ex vivo kidney image and in a parenchymal region free of embolized vessels, respectively. Each ROI was about 3500 voxels (1.5 mm^3^). In addition, we segmented the embolized kidney parenchyma. We recorded the mean CT attenuation values (HU) in each of these three areas and computed the signal-to-noise ratio (SNR) as SNR ROI = mean HU ROI/SD HU ROI, where SD is the standard deviation of the mean value [23,24,25]. To assess subjective image quality, we evaluated noise on a 5-point Likert scale (1, unacceptable noise; 2, above-average noise; 3, average noise; 4, less-than-average noise; 5, minimal noise). Disagreements were resolved by consensus [25,26].

### 2.4. Statistical Analysis

Continuous variables (e.g., SNR, indexed cast ratio and cast-to-capsule distance) were described as mean ± SD. As the three indexed cast ratio ranges were not independent from one another, we analyzed only a cast attenuation of ≥1300 HU to avoid overfitting. For qualitative and semi-quantitative variables, we computed the median [interquartile range]. Semi-quantitative variables (e.g., distality, cast-heterogeneity, and Likert image-quality scores) were handled as ordinal variables. Given the small number of images with a quality score of 5, we collapsed the groups with scores of 4 and 5 into a single group. Fragmentation and increased cortical blurring were dichotomous variables. The linearity of each variable was verified. Multivariate regression analyses were performed using a manual backward procedure. We used ordinal regression for ordinal variables, linear regression for quantitative variables, and logistic regression for dichotomous variables. Several intermediate models were required to develop the optimal model while confidently excluding potentially interesting variables such as NBCA concentration or rabbit weight. *p* values of 0.05 or less were considered significant. For increased cortical blurring, we made a ROC analysis given the high significativity of the variables in the model. Statistical analyses were performed using STATA software version 15.1 (STATA, College Station, TX, USA).

## 3. Results

### 3.1. Experiments

We studied 42 kidneys (26 right and 16 left) from 26 female rabbits weighing 2.53 to 4.38 kg. Mean injected glue-mixture volume per kidney was 0.81 ± 0.25 mL and mean injection time was 27.07 ± 9.00 s. BBF was used for 22 kidneys. 

For one kidney in the BBF group, the injection was stopped before backflow occurred at the microcatheter tip because fluoroscopy showed renal vein emboli. Ex vivo micro-CT visualized multiple arteriovenous fistulae at the corticomedullary junction. For complete details, see Table 2.

### 3.2. Cast Distribution Outcomes

#### 3.2.1. Objective Outcomes

**Figure 2:**
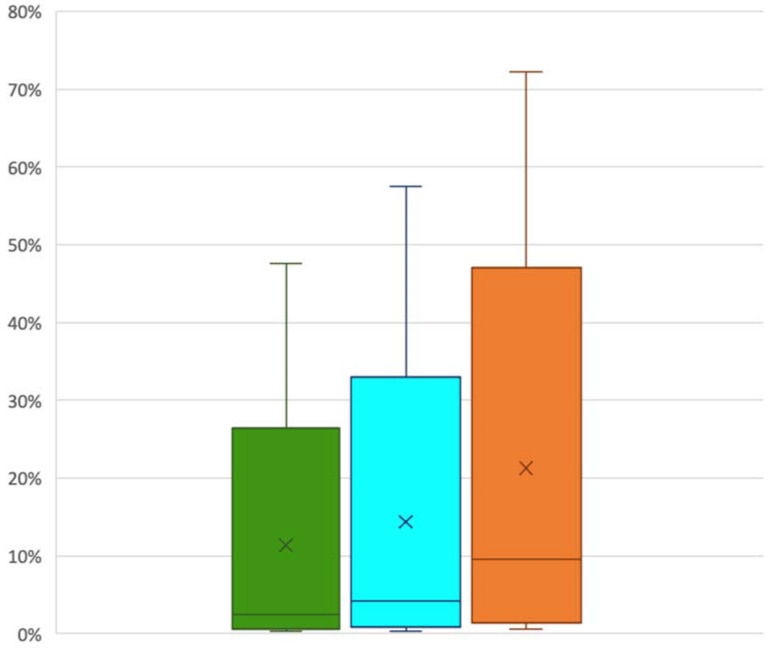
Cast percentage of segmented embolized kidney higher than 1600 HU (green column), higher than 1300 HU (blue column) and higher than 1000 HU (orange column).

The percentage of segmented embolized kidney containing cast varied with the indexed cast ratio considered (Figure 2). The highest observed value was 72% and the lowest was less than 1%. The mean cast-to-capsule distance varied from 0.09 mm to 5.04 mm. 

#### 3.2.2. Subjective Outcomes

Table 3 shows that, in 28 (67%) kidneys, the glue penetrated the superficial cortical interlobular arteries. However, only 19 (45%) kidneys exhibited increased cortical blurring indicating capillary penetration. The cast was fragmented in 24 (57%) kidneys. The mean cast-heterogeneity score was 2.57 ± 1.09 in the main renal artery and first branches, and 2.60 ± 0.91 in the interlobar arteries. 

**Figure 3:**
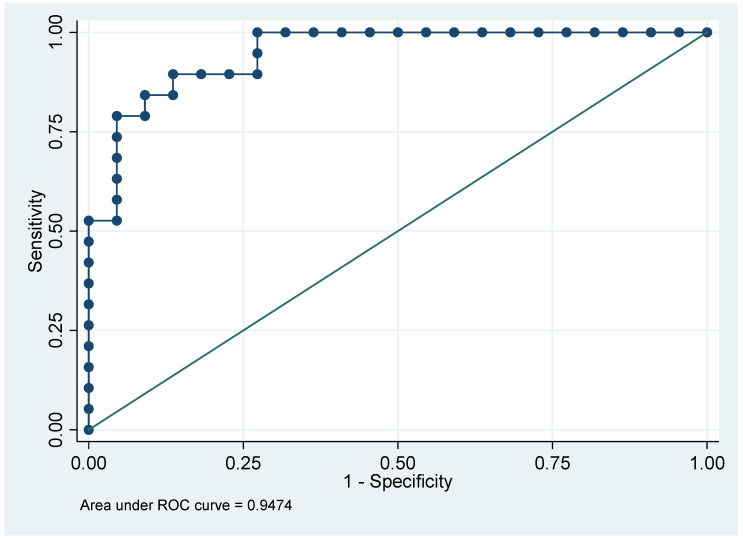
Receiver operating characteristics curve for the increased cortical blurring sign. This ROC curve displays the convenience of the cortical blurring sign considering independently associated covariates (found in the multivariate analysis).

Increased cortical blurring was highly correlated with all the other variables proving that studied parameters really impact the capillary bed penetration as shown by the receiver operating characteristics curve (Figure 3). 

### 3.3. Multivariate Analyses

A lower glue concentration was independently associated with greater distality of penetration (*p* = 0.000)—see Table 4. For cast attenuation and increased cortical blurring, the associations with greater distality were nearly significant. 

BBF was independently associated with greater distality (*p* = 0.000), no fragmentation (*p* = 0.009), deeper penetration within the renal cortex (*p* = 0.014), and a shorter cast-to-capsule distance (*p* = 0.002). Note that higher indexed cast ratio was not significatively correlated with BBF (*p* = 0.34). The injected glue-mixture volume showed independent positive associations with greater cast attenuation (*p* = 0.024) and deeper penetration into the renal cortex (*p* = 0.007). The use of AE was independently associated only with cast heterogeneity in zone 1. Higher animal weight was independently and negatively associated with deeper penetration. 

### 3.4. Image Quality

The mean SNRs of the background ROI, kidney ROI, and segmented parenchyma were 74.65 ± 104.67 (range, 4.85–361.3), 7.04 ± 2.23 (range, 4.25–15.25), and 2.91 ± 0.67 (range, 1.35–4.16), respectively (Table 5). The most common noise ratings on the Likert scale were 3 (19 [45%] kidneys) and 4 (17 [40%] kidneys). No images were rated 1 (unacceptable image noise). Table 6 reports the results of the multivariate analysis of image-quality variables. 

## 4. Discussion

In our study, we chose to focus on renal artery model for embolization because it is a terminal blood distribution. This enabled us to track the complete glue cast in the distal vascular network to better quantify its final place [27]. In our ex vivo micro-CT assessment of rabbit kidneys after in vivo renal-artery embolization with combined NBCA and LUF, this mixture penetrated to the deepest of the five arterial zones (superficial interlobular arteries) in about two-thirds of cases and into the capillaries in nearly half the cases. Cast fragmentation was seen in slightly more than half the specimens. Both greater NBCA dilution and BBF were independently associated with greater distality of penetration. Injection of a greater mixture volume was independently associated with higher indexed cast ratio and deeper penetration. Distality of penetration decreased as rabbit weight increased. Indexed cast ratio study did not show any significative association in the statistical model we made, but NBCA dilution and injected volume displayed a positive significant association. The chosen cutoffs may have influenced our results. The NBCA concentration and injection rate may vary with vessel diameter, blood-flow velocity, and distance from the microcatheter tip to the target [10,28]. Our objective evaluation of casts on micro-CT reconstructions showed that greater NBCA dilution by LUF was associated with the ability to embolize narrower arteries, in keeping with earlier data obtained using CT volumetry and not micro-CT as in our study [10]. Embolization extending distally to the capillary bed was assessed based on increased cortical blurring, which showed an independent association with BBF and injected volume, as well as a nearly significant association with greater NBCA dilution. The ROC curve confirmed the usefulness of this sign. Histological studies to document that increased cortical blurring reflects capillary-bed penetration and to differentiate influence on inflammation, and ischemia would be useful. The mean injected volume (including into the dead space) was 0.81 mL (0.50 mL–1.95 mL) in our rabbit model, compared to 0.43 mL (0.3–0.6 mL) in a dog model. We chose rabbits because they share with rodents the advantages of small size, ease of handling and housing, and relatively low cost compared to swine [15,29,30]. The species taking account corresponding coagulation parameters and vessel size may not substantially affect the behavior of NBCA mixtures, since polymerization is initiated and propagated by anions present in all mammals [18,31,32]. Note that polymerization and subsequent vessel occlusion can occur even in anticoagulated blood. BBF decrease cast fragmentation and heterogeneity, particularly in the interlobar renal arteries. In our work, BBF was associated with the absence of fragmentation, greater distality of penetration, and a shorter cast-to-capsule distance. It has potential clinical implications. Indeed, blocked blood flow technique may be used in some situations where deep penetration of the glue embolic agent is needed for better outcomes such as tumor devascularization or prostatic artery embolization for benign prostate hyperplasia. Furthermore, BBF allows for slower and more controlled delivery of the glue in the distal vascular bed depending on the injection pressure [2]. Balloon occlusion technique during glue embolization has the same advantage in clinical practice. It can be useful when the flow cannot be blocked by the microcatheter itself in order to prevent reflux but also to assist distal penetration. Moreover, a lower frequency of reflux without increased microcatheter adhesion has been demonstrated with BBF, which may be more likely to achieve continuous and dense casts even within an extensive vascular bed [13,33]. Although greater NBCA dilution was associated with greater distality of embolization, lower viscosity related to a higher NBCA concentration may be helpful if polymerization is markedly delayed flushing out 5% dextrose before using BBF. AE added to NBCA mixtures decreases adhesiveness and glue-induced sclerosis, while causing less tissue toxicity than does AE alone. In previous experimental studies, NBCA polymerization was accelerated by adding AE [34], and high proportions of AE in NBCA-LUF mixtures increased cast cohesion after injection without BBF [20]. A study in swine of various mixtures of NBCA, LUF, and AE used to pack wide-necked aneurysms demonstrated that an NBCA concentration of at least 30% was required to achieve embolization without migration [21]. In our study, the only variable independently associated with AE in the mixture was cast heterogeneity. To our knowledge, micro-CT has not been used previously to investigate NBCA embolization. Cast heterogeneity can be assessed by micro-CT, whereas the presence of thrombus or other material cannot. The cast is often heterogeneous when the injection is performed with preserved blood flow, due to variations over time in the polymerization rate [18,35].

In our model, the only variable independently associated with adding AE to the mixture was cast heterogeneity in the main renal artery. This finding is in contradiction to earlier studies [20,21,36,37]. Our study design may not have been appropriate for assessing links between AE and fragmentation, embolization distality, or injected volume. In clinical practice, angiography is used to guide embolization, and monitor the degree and extent of arterial occlusion [29]. Technical advances are leading to the increased use of high-resolution micro-CT [38]. For technical reasons, when small animals such as rabbits are studied, only ex vivo specimens can be evaluated by micro-CT [39]. Advantages of micro-CT include 3D reconstruction at micrometer resolution, ease of use, and a range of postprocessing options [38]. The main drawbacks are the use of ionizing radiation, possible occurrence of streak artifacts, and relatively low spontaneous contrast in soft tissues [40]. Image quality Likert scale and segmented renal parenchyma aera SNR are concordant considering significant factors associated. Both are entire appraisals of the image and that is probably why they corroborated each other. On micro-CT images, given the partial volume effect, the voxels adjacent to an artery are influenced by vessel density: with a smaller injected volume, the number of affected voxels is also smaller and the SNR is therefore higher. The wide variability of SNR values observed in the background ROI may be ascribable to the greater effect of streak artefacts in air than in the renal parenchyma. This possibility is consistent with the greater number of variables, including rabbit weight, independently associated with SNR in the renal ROI; the independent association with preserved blood flow during embolization may be related to more proximal NBCA polymerization compared to BBF. Considering these results, distality of the injection is linked to static parameters as the dilution, and to dynamic one as the blood flow. In our model, injected volume and rabbit weight cannot be useful for clinical practice but heterogeneity and fragmentation need to be modulated to limit secondary migration or non-targeted embolization. 

Our study has limitations. First, given the absence of previous data on micro-CT segmentation after embolization, we used arbitrary HU cutoffs. Second, the fact that AE addition was independently associated only with cast heterogeneity raises the possibility of limited reproducibility of the AE mixtures during the five to six repetitions of each experimental condition. Small sample size is another possible factor. Third, our use of an automatic injector to ensure standardization did not replicate clinical practice [27]. Moreover, differences exist between rabbits and humans in the coagulation and fibrinolysis systems, vessel diameters, intraarterial pressures, and other features of the vasculature. Finally, we studied healthy animals. For tumors, notably those with hypervascularization, alterations in the local microenvironment might affect the results of embolization [2,41].

## 5. Conclusions

Our ex vivo micro-CT study after in vivo embolization showed that objective and subjective parameters of glue cast can be conclusive with micro-CT. An important finding is that BBF was associated with greater distality of penetration, absence of cast fragmentation, and a shorter cast-to-capsule distance. This enabled physicians to be more ischemic for lesion vascularization with no target embolization (compare to microparticles). An increase in cortical blurring deserves further attention as a potentially useful sign for detecting capillary bed embolization.

Further work with histological studies in larger numbers of animals would be of interest. The use of larger animals such as swine, whose coagulation system is more similar to that of humans, would be expected to provide useful additional information. Studies focusing specifically on polymerization time and effects of 5% dextrose when using BBF would also be helpful.

## Figures and Tables

**Table 1:** Number of transarterial renal-artery embolization procedures with and without blocked-blood-flow (BBF) for each mixture of N-butyl cyanoacrylate and lipiodol, with or without anhydrous ethanol.

Ratios ^a^	BBF	Number of Procedures
1:3	No	5
1:3	Yes	5
1:1	No	5
1:1	Yes	5
1:7	No	5
1:7	Yes	6
1:2:1	No	5
1:2:1	Yes	6

^a^ ratio of NBCA (first number) over Lipiodol Ultra Fluid (second number) over anhydrous ethanol (third number); BBF, blocked blood flow.

**Table 2:** Experimental details for the 42 kidneys.

Variables	*n* (%) or Mean ± SD
Rabbit weight, kg, mean ± SD	3.18 ± 0.39
Side, *n* (%)	
*Right*	26 (61.90)
*Left*	16 (38.10)
Injected glue-mixture volume (including dead space), mL, mean ± SD	0.81 ± 0.25
Injection time (including dead-space flushing), s, mean ± SD	27.07 ± 9.00
Blocked blood flow, *n* (%)	22 (52.38)
Glubran^®^2 concentration, %, *n* (%)	
*12.5%*	12 (28.57)
*25%*	19 (45.24)
*50%*	11 (26.19)
Mixture containing 25% anhydrous ethanol, *n* (%)	11 (26.19)
Injection termination criterion reached, *n* (%)	41 (97.62)

*n*, number; SD, standard deviation; %, percentage.

**Table 3:** Subjective outcomes.

Variables	*n* (%) or Mean ± SD
Cast distality ^a^, *n* (%)	
*Zone 3*	7 (17)
*Zone 4*	7 (17)
*Zone 5*	28 (67)
Increased cortical blurring, *n* (%)	19 (45)
Cast fragmentation, *n* (%)	
*No*	19 (45)
*Yes*	23 (55)
Cast heterogeneity score ^b^	
*Renal artery and first branches*	2.57 ± 1.09
*Interlobar artery*	2.60 ± 0.91

^a^ zone 3, corticomedullary junction; zone 4, deep cortical interlobular arteries; and zone 5, superficial cortical interlobular arteries; ^b^ Only in the two most proximal zones (1 and 2); the score could range from 1 to 5.

**Table 4:** Multivariate analyses.

Variables	*p*-Value	95% CI
Subjective distality		
NBCA concentration	0.000	−17.3 to 5.24
Blood flow	0.000	−21.3 to 17.66
Glue-mixture volume injected (including into dead space)	0.263	−5.45 to 19.97
Rabbit weight	0.093	−2.76 to 0.21
Cast fragmentation		
NBCA concentration	0.437	−7.00 to 3.02
Blood flow	0.009	0.56 to 4.04
Glue-mixture volume injected (including into dead space)	0.250	−19.60 to 5.11
Increased cortical blurring		
NBCA concentration	0.068	−18.81 to 0.66
Blood flow	0.014	−4.55 to 0.51
Glue-mixture volume injected (including into dead space)	0.007	3.77 to 24.20
Rabbit weight	0.018	−6.34 to 0.60
Anhydrous ethanol in the mixture	0.103	−0.34 to 3.73
Cast heterogeneity Zone 1 ^a^		
*Blood flow*	0.077	−2.54 to 0.13
*Glue-mixture volume injected (including into dead space)*	0.073	−0.41 to 9.40
*Anhydrous ethanol in the mixture*	0.048	−2.81 to 0.01
Zone 2 ^b^		
*NBCA concentration*	0.244	−7.17 to 1.82
*Blood flow*	0.005	−3.59 to 0.62
*Glue-mixture volume injected (including into dead space)*	0.066	−0.27 to 8.29
*Rabbit weight*	0.202	−0.50 to 2.38
Cast-to-capsule distance (mean)		
NBCA concentration	0.127	−0.69 to 5.22
Blood flow	0.002	0.49 to 1.92
Glue-mixture volume injected (including into dead space)	0.209	−2.10 to 0.48
Indexed cast ratio		
Glue concentration	0.023	−0.25 to −0.02
Blood flow	0.339	−0.05 to 0.02
Glue-mixture volume injected (including into dead space)	0.000	0.35 to 0.50
Rabbit weight	0.187	−0.08 to 0.02
Anhydrous ethanol in the mixture	0.584	−0.05 to 0.03

^a^ Main renal artery and first branches; ^b^ Interlobar arteries; 95% CI: 95% confidence interval.

**Table 5:** Image quality data.

Variables	*n* (%) or Mean ± SD
Likert scale rating, *n* (%)	
*2*	5 (12)
*3*	19 (45)
*4*	17 (40)
*5*	1 (2)
SNR, mean ± SD	
*Background ROI*	74.65 ± 104.67
*Kidney ROI*	7.04 ± 2.23
*Segmented kidney parenchyma*	2.91 ± 0.67

SNR, signal-to-noise ratio; ROI, region of interest.

**Table 6:** Multivariate analysis of image-quality variables.

Variables	*p*-Value	95% CI
Likert scale rating		
Glue-mixture volume injected (including into dead space)	0.015	−6.31 to 0.69
Rabbit weight	0.153	−0.51 to 3.26
SNR in background ROI		
Blood flow	0.666	−56.19 to 86.41
Rabbit weight	0.054	−129.99 to 1.10
Anhydrous ethanol in the mixture	0.272	−113.66 to 33.39
SNR in renal ROI		
NBCA concentration	0.401	−1.90 to 4.60
Blood flow	0.004	0.97 to 4.51
Glue-mixture volume injected (including into dead space)	0.094	−0.31 to 3.69
Rabbit weight	0.032	−4.36 to 0.21
Anhydrous ethanol in the mixture	0.059	−0.06 to 2.77
SNR in segmented parenchyma		
Blood flow	0.281	−0.20 to 0.67
Glue-mixture volume injected (including into dead space)	0.000	−1.68 to 0.60
Anhydrous ethanol in the mixture	0.193	−0.14 to 0.64

95% CI, 95% confidence interval; SNR, signal-to-noise ratio; ROI, region of interest.

## Data Availability

All the study data are reported in this article.
